# Asymmetrical glymphatic dysfunction in patients with long Covid associated neurocognitive impairment- correlation with BBB disruption

**DOI:** 10.1186/s12883-025-04133-4

**Published:** 2025-03-19

**Authors:** Joga R. Chaganti, Tanush K. Talekar, Bruce James Brew

**Affiliations:** 1 Thomas Jefferson University Hospital, Philadelphia, PA 19107 USA; 2 Advanced Post-Processing Laboratory For Functional MRI and DTI, The Jefferson Integrated Magnetic Resonance Imaging Center (JIMRIC), Thomas Jefferson University, Philadelphia, PA 19107 USA; 3 University of New South Wales, Sydney, Australia; 4 Head Neurosciences Program and Peter Duncan Neurosciences Unit, St Vincent’s Centre for Applied Medical Research, Sydney, Australia; 5 Department of Neurology, St Vincent’s Hospital, Sydney, Australia

**Keywords:** PASC-CI, MRI, DTI-ALPS, BBB, KTRANS

## Abstract

**Background and purpose:**

The glymphatic system, a waste clearance pathway, has been implicated in several neurological conditions associated with neuroinflammation. COVID-19 associated neurocognitive impairment, part of the post-acute sequelae of SARS-CoV-2 infection (PASC), is strongly associated with neuroinflammation and disrupted blood-brain barrier (BBB). Several studies have implicated a synergistic interaction between the glymphatic system dysfunction and BBB disruption. In this proof-of-concept study, we investigated the role of the MRI metric diffusion along the perivascular spaces DTI (DTI-ALPS) in patients with PASC and correlated this with the BBB capillary permeability metric- K trans derived from Dynamic contrast enhanced (DCE) perfusion.

**Materials and methods:**

14 subjects with PASC who had persisting symptoms of anosmia, ageusia, fatigue, and cognitive impairment (CI) and ten healthy age and sex matched controls were recruited. All PASC subjects underwent routine and advanced MR brain imaging at two time points, (3 months +/- 2 weeks) after initial infection - referred as Time Point 1 (TP-1) - and 10 repeated the MRI scan 12 months (+/- 2 weeks) later - referred as Time Point 2 (TP-2), while the controls had MR imaging done only at TP-1. All had mild neurocognitive impairment. In the final analysis we included those who had DTI study at both time points (n-10). MR imaging included DCE perfusion and DTI in addition to anatomical imaging.

**Statistical analysis:**

Given the small size of the sample and nonnormality of data in the descriptive analyses, nonparametric analyses were used for group comparisons. A two-sample Wilcoxon rank sum test was used to show the differences in DTI-ALPS between the patients and controls in the predefined regions of interest. Spearman’s correlation coefficient (rho) was used to assess the correlation between DTI-ALPS index with K trans.

**Results:**

There was significant reduction in the DTI-ALPS index between the patients and controls in the left hemisphere (z = 2.04, *p <* 0.04). However, there was no significant change over time in the index. There was a strong inverse correlation between the central white matter K trans and DTI-ALPS index (rho = 0.66, *p* < 0.03).

**Conclusion:**

Our study indicates that disordered para vascular drainage, a marker for glymphatic system and BBB damage may contribute to neurocognitive impairment (NCI) among patients with PASC. The DTI-ALPS index, which does not require contrast injection, has the potential to serve as a non-invasive biomarker.

**Supplementary Information:**

The online version contains supplementary material available at 10.1186/s12883-025-04133-4.

## Introduction

Cognitive impairment (CI) is one of the common symptoms of post-acute SARS CoV-2 infection (PASC), otherwise known as long covid [1]. Existing evidence indicates neuroinflammation is one of the important driving forces that is responsible for CI in patients with PASC. Several studies have reported that PASC is associated with a disrupted blood-brain barrier (BBB), and neuroinflammation [2, 3]. Further, there is imaging evidence of BBB impairment through increased capillary permeability [4] likely leading to neuroglial vascular unit dysfunction. These observations are similar in patients with several neurocognitive disorders such as Alzheimer’s disease where increased permeability is also associated with decreased clearance of solutes [5]. New insights indicate that this solute clearance is a function of glymphatic system [6], which is an alternate pathway of the scavenger system in the brain that facilitates the exchange of the metabolites between the perivascular space CSF and the brain. This is a brain-wide pathway for fluid transport, possibly starting as the para-arterial influx of subarachnoid CSF into the brain interstitium, followed by the clearance of interstitial fluid (ISF) along large-calibre draining veins as well as through the basal foramina and from there on to cervical lymphatics [7]. The glymphatic system has been investigated in vivo using dynamic contrast-enhanced MRI, intrathecal administration of gadolinium [8] and dynamic 11 C-Pittsburgh Compound B positron emission tomography techniques [9]. Recently, diffusion MRI has been proposed as a non-invasive method to quantify glymphatic function by calculating the diffusion tensor image metrics along the perivascular space (DTI-ALPS) index [10]. The DTI-ALPS index appears to be correlated with more classical detection methods of glymphatic clearance function [8]. This method has been applied in studies on Alzheimer’s disease, Parkinson’s disease, ischemic stroke, sleep, idiopathic normal pressure hydrocephalus, tumor-associated cerebral edema and idiopathic intracranial hypertension [11–15].

The BBB disruption in neurodegenerative disorders is at an extremely low level below current detection limits using standard MRI techniques. However, recent advanced MRI methodologies such as DCE-Perfusion (DCE-P) MRI have proven to be robust enough to identify subtle BBB disruption. DCE-P MRI enables the calculation of a novel parameter K trans, which calculates the efflux of gadolinium from blood plasma to the extravascular-extracellular space [16]. The K trans measurement depends on blood flow (F), vascular permeability (P), and surface area (S). A meta-analysis of K trans measurement has validated its utility in identifying BBB impairment in a variety of neurological conditions [16]. The metrics of BBB disruption can be obtained using different models. At a very low level of disruption of the BBB, the two-compartment Patlock model is considered to be the most robust method to generate reproducible K trans results [17].

In this “proof-of-concept” study, our primary objective was to investigate the relationship of the metric diffusion along the perivascular spaces derived from DTI (DTI-ALPS) in subjects with PASC at two time points. In the secondary analysis, we explored the relationship of DTI-ALPS and K trans. We hypothesized that increased capillary permeability through BBB impairment (measured by K trans) would be associated with reduced clearance of the solutes from the interstitial space (measured by DTI-ALPS). If true this would enable repeated MRI scans without the concern over contrast administration.

## Materials and methods

14 subjects with PASC who had persisting symptoms of anosmia, ageusia, fatigue, and CI who were clinically evaluated at the Neurology Department at SVH between July 2021-August − 2022 were consented for the use of their clinical data for research. All 14 patients had been referred for evaluation of CI post COVID and all underwent a full neurological assessment (BJB). All the subjects were right-handed. All participants underwent routine and advanced MR imaging early in the disorder (3 months +/- 2 weeks after initial infection) - Time Point 1 (TP-1) and 10 repeated the MRI scan later (12 months (+/- 2 weeks) - Time Point 2 (TP-2). Hence in the final analysis we only included those who had DTI studies at both time points (n-10). Seven of those participants has been enrolled into the ADAPT study, a prospective cohort of 128 SARS-CoV-2 positive patients and had received serial measurements of cognition with the Cogstate Brief Battery [1]. Individuals with a prior history of drug use, significant head injury, psychiatric illness, and hepatitis C virus co-infection were excluded. Ten healthy age and sex matched controls were recruited and underwent the same neurological assessments at one time point (Table 1). Local ethics approval was obtained from St Vincents Hospital, Sydney, Australia, Hospital Human Research Ethics Committee- (Approval 2022/ETH0022). All the participants provided written informed consent prior to enrolment. The study was conducted in adherence to the principles outlined in the Declaration of Helsinki and relevant regulatory guidelines.

**Table 1:** Baseline Characteristics among PASC CI cases and controls

Baseline characteristics among PASC CI cases and controls		
	Cases (*n* = 10)	Controls (*n* = 10)
Sex		
Male	4 (43%)	5 (50%)
Female	6 (57%)	5 (50%)
Age (years, mean and SD)	49 (+/-2)	46 (+/-1.7)
Duration between COVID-19 diagnosis and first MRI (weeks, mean and SD)	12 (+/- 1)	N/A
Acute Covid Severity		
Mild	6 (78.9%)	
Moderate	3 (14.2%)	
Severe	1 (7.1%)	
Neurological Symptoms		N/A
Loss of Smell	9 (90%)	
Loss of Taste	8 (80%)	
Cognitive difficulty	10 (100%)	
Myalgias	10 (100%)	
Neurology at TP-1		N/A
Loss of Smell	10 (100%)	
Loss of Taste	10 (100%)	
Fatigue	10 (100%)	
Cognitive difficulty	10 (100%)	

Imaging was performed with a 3TMR imaging scanner (Ingenia; Philips Healthcare, Best, the Netherlands) with a 24-channel head coil. DCE perfusion imaging, 32 directional diffusion imaging and single voxel in addition to the routine clinical imaging (T-1 volumetric imaging and T-2 FSE) were performed.

T1-weighted imaging was performed with the following parameters 3DT-1 spoiled gradient recalled acquisition in steady state (SPGR): 128 sagittal slices, 1 mm isotropic, time to repeat/time to echo (TR/ TE): shortest, field of view: 240, Matrix: 256/256.

### Diffusion tensor imaging (DTI)

The DTI protocol consisted of a single-shot spin-echo-based echo- planar diffusion-weighted imaging with three averages and 36 gradient encoding directions, with b values of 0 and 1,000 s/mm2. The imaging parameters were slice thickness 5 mm, interslice gap 1.5 mm, FOV: 230 × 230, matrix 128/128, TR 3500 and TE 96Msec.

### Image processing

#### DCE perfusion MRI

The DCE-MRI sequence was obtained using 3D T1- weighted spoiled gradient echo sequence in the axial plane covering the entire brain [TR and TE⁄ shortest], flip angle 15^0^, FOV 230/230, voxel size 1 × 1 × 1 mm, with number of signal averages = 1, number of dynamics = 90 and scanning time = 9.06 min. Contrast injection was commenced 6 s after the start of the dynamic MRI acquisitions, given in the form of a bolus injection of Gadobutrol (Gadovist, Bayer, California, USA) at a concentration of 0.1 mmol/kg of body weight at 3 ml/s. Following the DCE-MRI scan, postcontrast- enhanced volumetric T1-weighted images were acquired as part of the routine clinical examination.

### Image processing

#### DTI and DTI-ALPS

The DTI was processed using FSL toolbox (https://fsl.fmrib.ox.ac.uk/fsl/fslwiki). FSL’s eddy correction tool was used for pre-processing the data. The brain extraction tool (BET) was used to create brain masks. Subsequently, water diffusivity along the x (Dx), y(Dy), and z (Dy) axes and fractional anisotropy (FA) maps were computed for each DTI scan. The tract-based skeleton statistic (TBSS) was used for the registration of FA maps from each participant onto an MNI FA atlas. All diffusivity maps were aligned to the same space using TBSS non-FA scripts. This method extracted affine matrices and warp fields derived from FA registration and applied them to these diffusivity maps.

**Fig. 1:**
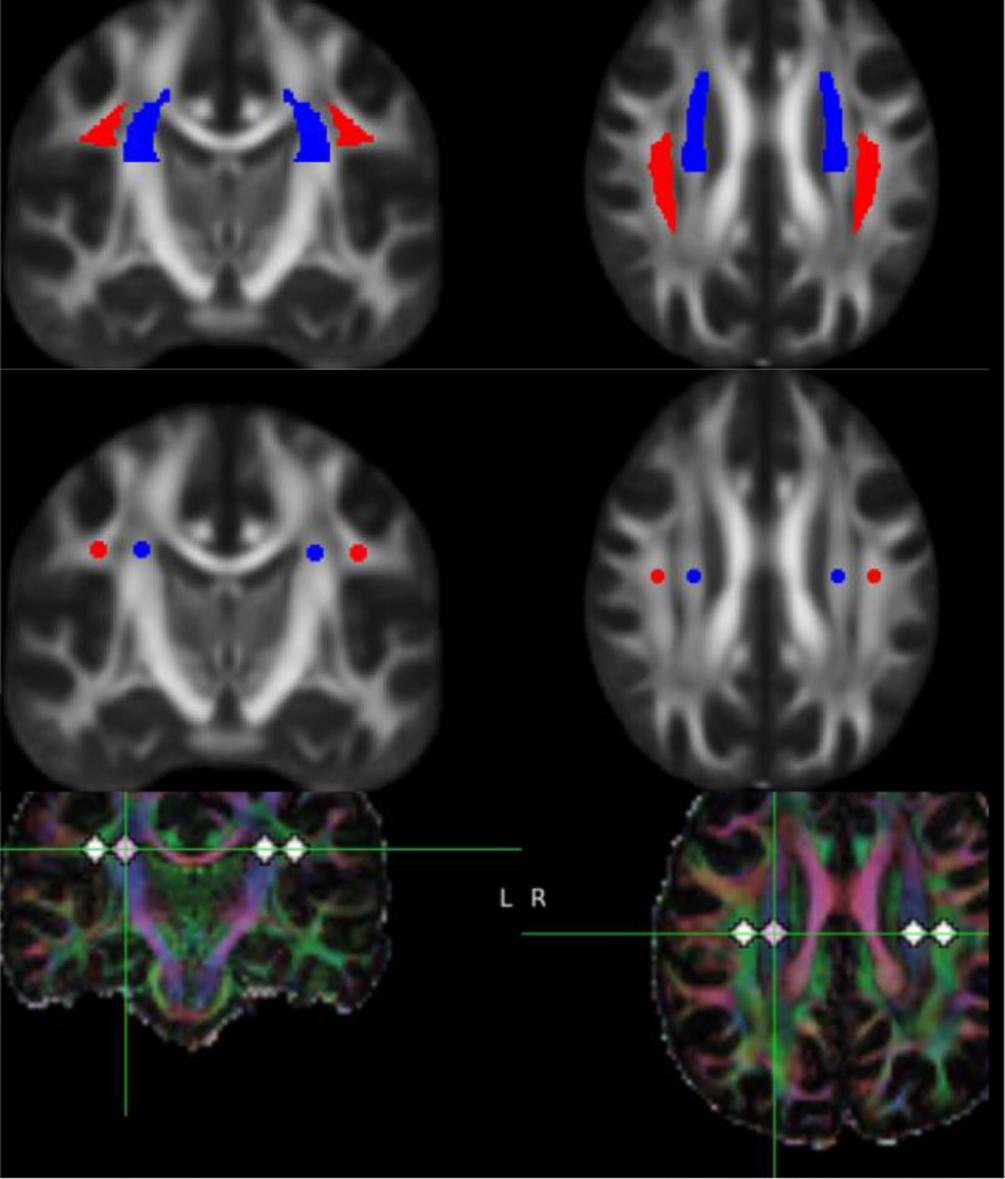
DTI-ALPS with ROI localisation. DTI Raw data in both directional color coding and gray scale showing placement of ROI in projection and association fibers roughly at the point where they are roughly perpendicular to each other. ROI are chosen both manually and automated segmentation methods

To calculate the DTI-ALPS, regions of interest (ROI) were defined as 3 mm x 3 mm rectangle ROIs. The ROI were placed in the projection- and association-fiber regions in the horizontal plane of the lateral ventricle body. Three ROIs were placed in bilateral regions (Fig. 1).

The DTI-ALPS is calculated as:

$${\rm{DTI - ALPS = }}{{{\rm{(mean}}\,{\rm{(Dx}}\,{\rm{(proj),}}\,{\rm{Dy}}\,{\rm{(assoc)))}}} \over {{\rm{(mean}}\,{\rm{(Dy(proj),}}\,{\rm{Dz}}\,{\rm{(assoc)))}}}}$$

Here, Dx (proj) and Dy (assoc), Dz (assoc) are the mean diffusivity in the ROI placed in projection fibers and the association fibers along the x-axis, y-axis, and z-axis, respectively.

The DTI-ALPS values were calculated for each patient and controls at baseline and patients at the longitudinal timepoint.

#### DCE perfusion MRI

**Fig. 2:**
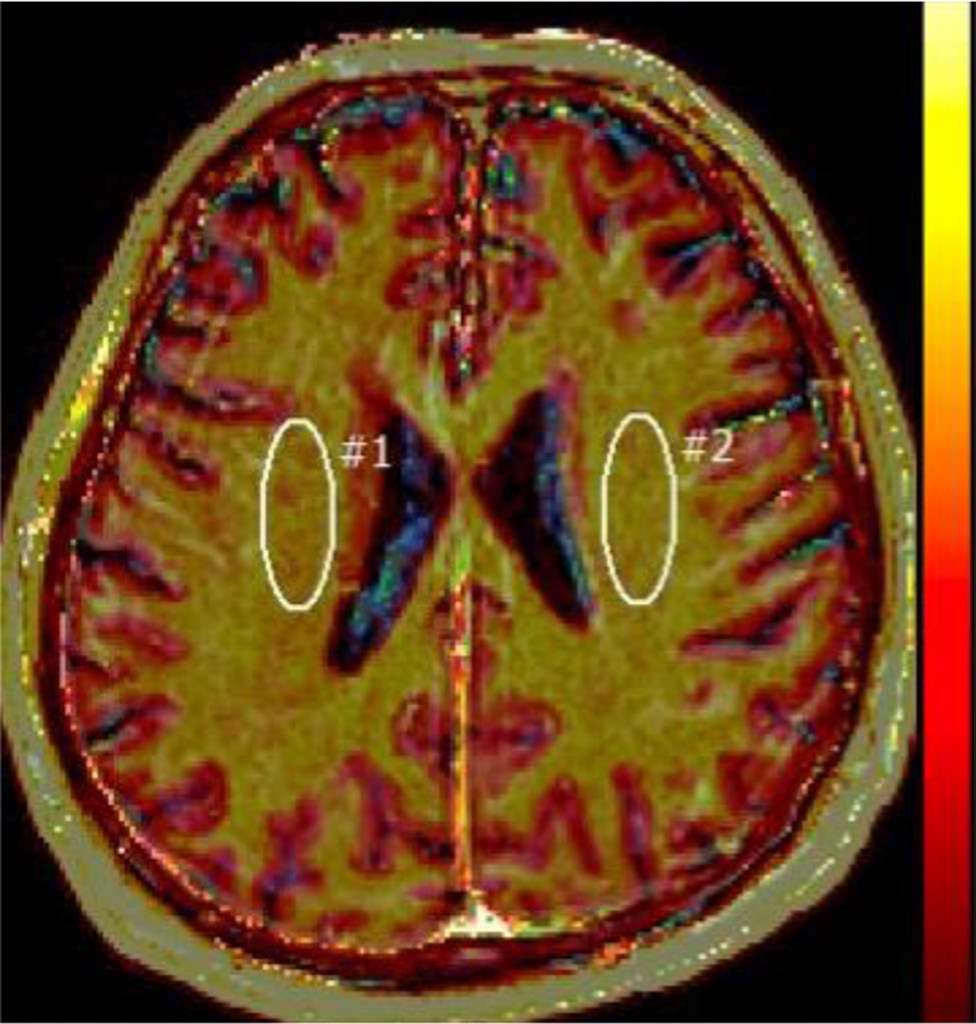
K Trans with ROI in the central white matter. DCE Perfusion using NordicICE with ROI in the central white matter

**Fig. 3:**
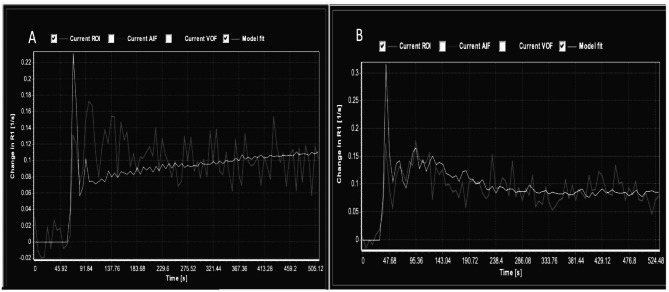
K trans Plots. DCE derived mean K trans Maps with localization in the centrum Semiovale A: Subjects; B: Controls, demonstrating signal intensity (SI) over time in subjects and in controls. Please note model fit curve is displayed in bright color and the raw values in greyish shade.

Dynamic contrast enhanced perfusion DCE studies were processed with nordicICE [NordicICE (NICE) 4.0.4; NordicNeuroLab, Bergen, Norway], a propriety software that includes brain extraction, motion correction and image registration. NordicICE is a proprietary software and was employed to measure the DCE-derived metric K trans. The software has inbuilt features to measure the baseline T-1 values of the tissue, to correct the leakage correction and removal of negative slope values (values below zero), which were used to offset the blood plasma volume intercept. A two-compartment pharmacokinetic model was applied in the region of interests by using the Patlak graphical approach based on linear fitting of scatter plots [12, 13], which was found to be the most appropriate model in a low-leakage regimen [12, 13]. This Patlak graphical approach provided the BBB leakage rate and the local blood plasma volume. The slope of this fit is the BBB leakage rate (assuming a tissue density of 1 g/ml), and the intercept is the local blood plasma volume. All the patients were tested for fit of the model (chi-square goodness of model fit). Moreover, at very low-level BBB disruption, the flow is not a significant contributor to K trans (cf. to enhancing MS plaques and tumours) [24]. The K-trans images were interrogated by placing multiple regions of interest (ROI) in the following areas of the brain (Frontal white matter, and cortex, Basal ganglia, Central white matter, by a radiologist with 30 years of experience (JC) Image analyst with I year experience (TT)). The volumes of the regions of interest were variable with larger volumes in the centrum Semiovale up to 5 Cm and in the lower level of 0.5 Cm area and DCE derived mean K trans maps were obtained (Figs. 2 and 3). The volumes of the regions of interest -the ROI was adjusted to reduce the effects of CSF.

### Statistical analysis

Out of 14 patients, only 10 patients had follow-up DTI study and hence in the final analysis we included those who had DTI study at both time points (n-10). Given the small size of the sample and nonnormality of data in the descriptive analyses, nonparametric analyses were used for group comparisons. For the primary endpoint, a two-sample Wilcoxon rank sum test was used to show the differences the differences in DTI-ALPS between the patients and controls in the predefined ROIs. Paired Wilcoxon rank sum test was used to compare the DTI-ALPS values between two time points and within the regions of interest (Both hemispheres). In the second analysis, we assessed whether there was a correlation between the BBB disruption metric K trans and DTI-ALPS using Spearman’s correlation coefficient (rho). The Levene test was conducted to examine the homogeneity of variance between the groups.

## Results

### DTI-ALPS

**Fig. 4:**
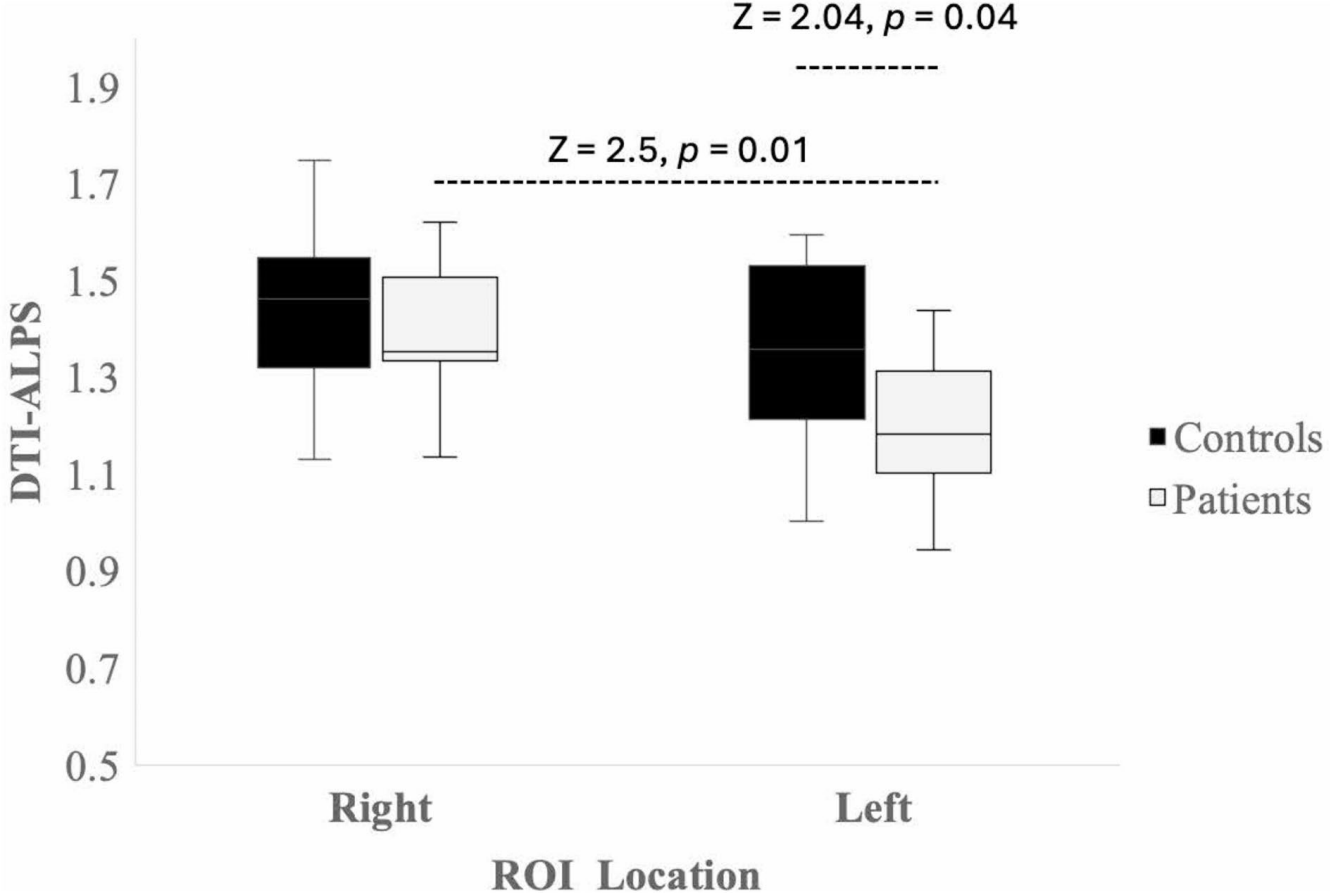
Bar graph depicting the results of Wilcoxon rank sum test. Wilcoxon rank sum test showing differences in DTI-ALPS in the left hemisphere and between right and left hemispheres

There was a significant difference (z = 2.04, *p* = 0.04) in DTI-ALPS between patients and controls in the left hemisphere. However, there was no statistically significant change (*p* > 0.05) over time within patients in this ROI. There was a significant difference in DTI-ALPS between Left and Right ROI within patients (z = 2.5, *p* = 0.01) (Fig. 4).

### Correlation with K trans and Levene test-retest measurement

The Levene test -retest statistic was 0.4919, with 2 degrees of freedom for the between groups and 27 degrees of freedom for the within groups, resulting in a p-value of 0.6168. The obtained p-value was greater than 0.05, indicating that there is no significant difference in variance between the groups.

**Fig. 5:**
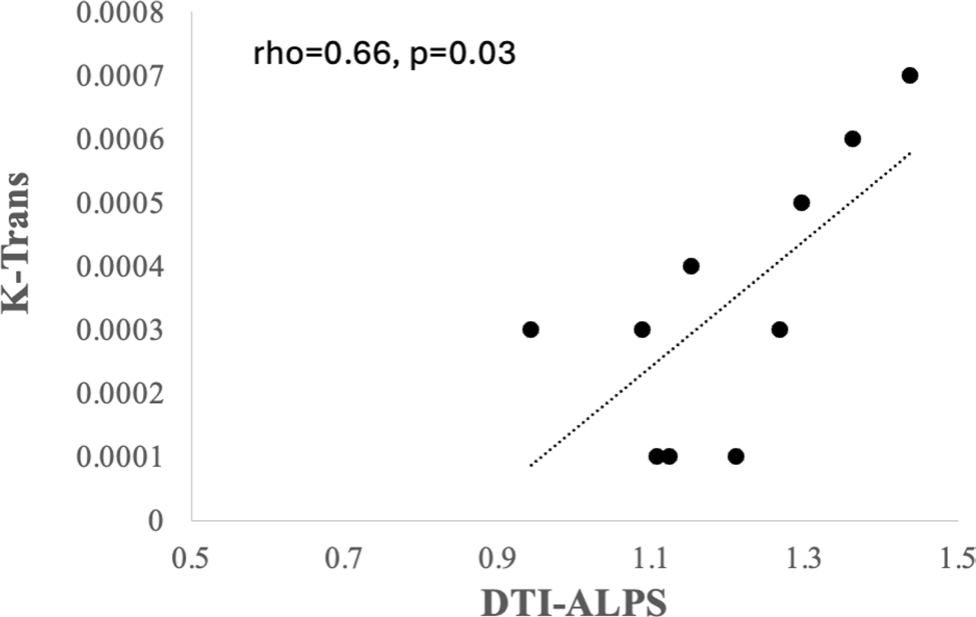
Correlation between the K trans and DTI-ALPS. Spearman’s correlation coefficient Scatter plot between the DTI-ALPS on X axis and K trans of the central white matter on y axis

Exploratory correlations were performed between K-trans, ALPS index. Mean K trans scores of the whole brain did not correlate with ALPS scores. However central white matter K trans was positively correlated on the side of abnormal ALPS (rho = 0.66, *p* < 0.03) (Fig. 5).

## Discussion

In this proof-of-concept prospective case-control study, we investigated glymphatic system abnormalities, as expressed by the DTI-ALPS index, and correlated them with BBB capillary permeability, as determined by K trans in individuals affected by cognitive impairment (CI) due to PASC. We found decreased DTI-ALPS index in the left hemisphere [1.383 (SD 0.202)], but not in the right hemisphere which remained within normal limits [1.47 (SD 0.1374)] as noted in the literature [18–20] (Fig:4). We observed a strong negative correlation between the DTI-ALPS index and K trans in the central white matter suggesting influx of potential toxins through an impaired BBB and decreased efflux clearance of these through an impaired glymphatic system.

We believe our study is the first to delve into the relationship between DTI-ALPS and the BBB metric K trans. Our own prior research has revealed disruptions in the BBB, likely triggered by glutamatergic excitotoxicity, and subsequent changes in white matter integrity among patients with PASC/long COVID [4]. BBB disruption is a known characteristic of several neurodegenerative disorders, often occurring alongside abnormalities in the glymphatic system, a crucial paravascular drainage pathway in the brain.

To our knowledge there is only one other study of ALPS in PASC/long Covid. Wu et al. showed decreased ALPS changes but their study was not longitudinal. This study concluded that the glymphatic dysfunction is most likely driven by neuronal inflammation.

Several earlier studies have identified excitotoxicity as a primary pathological mechanism contributing to CI in PASC/long COVID [21]. The excitotoxicity, in addition to causing BBB disruption, is also known to impair aquaporin-4 water channels [4, 22]. This dysregulation of water channels has been shown to result in impaired cerebrospinal fluid (CSF) influx and disruption of the CSF-interstitial fluid (ISF) turnover pathway [23] and thus appears to be having a synergistic relation with BBB disruption.

Our finding of an asymmetric reduction in the ALPS index in the left hemisphere compared to the right was also noted by Wu et al. [14]. Previous studies using the ALPS index in other diseases have only focussed on the dominant hemisphere since it is known that diffusion metrics differ from the non-dominant hemisphere [24].

Additionally, we also found that Dx- values in PASC/long Covid were mildly decreased compared to controls, leading to a decrease in the DTI-ALPS index (trending significance with variance between the controls and patients is 30.3% on polynomial regression). This suggests that diffusion is hindered more significantly in projection fibers than in association fibers, particularly in the plane perpendicular to the ventricles, possibly due to increased extracellular water content and changes in white matter fiber structure. The lack of significant change in the ALPS index over 12 months suggests that alterations contributing to the pathogenesis of altered drainage may take longer to revert or may be irreversible, a finding also reflected in the lack of statistically significant change in K trans values.

Despite the valuable insights provided by our study, several limitations exist, primarily the small sample size, partially compensated by the longitudinal design. Future studies with larger sample sizes and longitudinal designs are warranted to further elucidate these findings.

## Conclusion

Our study suggests that PASC/long covid CI is at least in part related to BBB impairment allowing influx of potential toxins and impaired glymphatics impairing appropriate efflux of such toxins. DTI-ALPS could serve could as an important non-invasive biomarker to identify these para vascular drainage abnormalities.

## Electronic supplementary material

Below is the link to the electronic supplementary material.

Supplementary Material 1

Supplementary Material 2

## Data Availability

The data will be made available on reasonable request from corresponding author and shared on preserved on repository.

## Abbreviations

PASC: Post-acute sequelae of SARS-CoV-2 infection
DTI-ALPS: Diffusion along the perivascular spaces DTI (DTI-ALPS)
NCI: Neuro cognitive impairment
BBB: Blood brain barrier
ROI: Regions of interest
SVH: St Vincent’s Hospital
